# Gain modulation and odor concentration invariance in early olfactory networks

**DOI:** 10.1371/journal.pcbi.1011176

**Published:** 2023-06-21

**Authors:** Emiliano Marachlian, Ramón Huerta, Fernando F. Locatelli

**Affiliations:** 1 Instituto de Fisiología Biología Molecular y Neurociencias (IFIByNE-UBA-CONICET) and Departamento de Fisiología, Biología Molecular y Celular, Facultad de Ciencias Exactas y Naturales, Universidad de Buenos Aires, Buenos Aires, Argentina; 2 Departamento de Física, Facultad de Ciencias Exactas y Naturales, Universidad de Buenos Aires, Buenos Aires, Argentina; 3 BioCircuits Institute, University of California San Diego, La Jolla, California, United States of America; University of Groningen, NETHERLANDS

## Abstract

The broad receptive field of the olfactory receptors constitutes the basis of a combinatorial code that allows animals to detect and discriminate many more odorants than the actual number of receptor types that they express. One drawback is that high odor concentrations recruit lower affinity receptors which can lead to the perception of qualitatively different odors. Here we addressed the contribution that signal-processing in the antennal lobe makes to reduce concentration dependence in odor representation. By means of calcium imaging and pharmacological approach we describe the contribution that GABA receptors play in terms of the amplitude and temporal profiles of the signals that convey odor information from the antennal lobes to higher brain centers. We found that GABA reduces the amplitude of odor elicited signals and the number of glomeruli that are recruited in an odor-concentration-dependent manner. Blocking GABA receptors decreases the correlation among glomerular activity patterns elicited by different concentrations of the same odor. In addition, we built a realistic mathematical model of the antennal lobe that was used to test the viability of the proposed mechanisms and to evaluate the processing properties of the AL network under conditions that cannot be achieved in physiology experiments. Interestingly, even though based on a rather simple topology and cell interactions solely mediated by GABAergic lateral inhibitions, the AL model reproduced key features of the AL response upon different odor concentrations and provides plausible solutions for concentration invariant recognition of odors by artificial sensors.

## Author summary

Animals can discriminate and recognize milliards of different odors. This is possible because odor detection relies on generalist olfactory receptors that together generate a powerful combinatorial code. A drawback is that high odorant concentrations can activate lower affinity receptors and this change in the combination of receptors can theoretically drive the perception of qualitatively different odors. In this context, concentration invariance, the ability of animals to recognize the same odor along a wide range of intensities is studied with neurobiological and computational interest. In the present study we combine calcium imaging experiments, pharmacology and computational modeling of the honey bees antennal lobe to unveil the neural mechanisms that allow animals to recognize an odor regardless of its concentration. The calcium imaging technique provided us the possibility to test not only the stability in intensity of odor-elicited signals but also the stability of the glomerular code that is needed to identify an odor. This last property has often been proposed as a natural consequence of the first one, but it is proven for the first time in this manuscript. In both, experiments and model, we prove that lateral inhibitions mediated by GABA-A and GABA-B receptors, contribute to reduce de impact of the concentration on the glomerular pattern that represents the odor.

## Introduction

Olfactory stimuli are basically described at the physical domain along two main dimensions; its chemical composition normally referred to as “odor quality”, and its concentration normally referred to as “odor intensity”. Importantly, because of the way in which odors are detected and encoded, both dimensions are not independent at the neural domain, which may cause that different concentrations of the same odorant are perceived as qualitatively distinct odors. In this context, odor concentration invariance, the ability of animals to recognize an odor across concentrations, is studied with neurobiological interest and as a generic theoretical problem with implications in object recognition technologies [1–4].

Several functional and neuroanatomical principles of the olfactory system are repeated across species [5–6]. One of them is that olfactory receptors are in general not specific to a single odorant, but can interact with a variety of them [7]. Second, each olfactory receptor neuron (ORN) expresses only one type of olfactory receptor, and therefore ORNs adopt the odor tuning determined by the receptor [8]. Third, all ORNs that express the same receptor send axons that converge into anatomically discrete subareas called glomeruli in the insect antennal lobe (AL) or in the vertebrate olfactory bulb (OB) [9–10]. These three characteristics produce that each odor elicits a combinatorial pattern of active glomeruli in the AL or OB that constitutes its primary representation. Importantly, the higher the odor concentration, the greater the probability that low-affinity receptors will be activated, producing a different primary representation [11–12]. In this context, the mechanisms that contribute to stabilize odor representation across concentrations and the role of GABAergic inhibition in the olfactory circuit are studied in insects [13–17], and vertebrates [18–20]. Inhibitory GABAergic local neurons in the AL and the OB form an intricate network of lateral and feed-forward interactions that play a critical role in processing and encoding of the olfactory information [21–24]. It is established that local GABAergic inhibition is critical for odor segregation, fast and slow temporal patterning of the AL and OB output activity, and gain control in the olfactory circuit [12,25–28].

Honey bees have rich social and individual behaviors that depend of their ability to recognize innate and learned odors [29,30]. Several functional and anatomical aspects of the olfactory circuit in honey bees have been described, and neural activity patterns elicited by odors can be measured along the olfactory circuit thanks well established preparations suited for electrophysiology and calcium imaging [31–34]. These characteristics make it an interesting model system in which behavior, physiology and computational modeling can converge to disentangle the neural mechanisms involved in olfactory processing [35]. Previous computational approaches have modeled the network topologies and the balance between excitatory and inhibitory interactions that are necessary to provide the honey bee AL properties that support complex computational challenges such as contrast enhancement between odors [36], identity and temporal resolution in complex mixtures [37–40] and experience-dependent plasticity [41,42]. Moreover, in regards to concentration dependence, it has been shown that addition of gain control implemented by lateral and feedback inhibitory interactions in the AL improves identity coding [35]. However, whether it is possible that absolute gain control emerges in the AL upon realistic changes in odor concentration is still debated [43].

Here we combine experimental and modeling approaches to investigate the contribution that GABAergic inhibition makes to attenuate the effect of concentration in odor representation in the honey bee antennal lobe. Odor elicited signals were measured by calcium imaging of the AL and analyzed in terms of intensity, number of recruited glomeruli and stability of the glomerular pattern that encodes the odor. This is to our knowledge, the first time that the contributions of GABA-A and GABA-B receptors are systematically measured in relation to the effect of odor concentration on the ensemble of glomeruli that encodes odor identity. In addition, we generated a realistic mathematical model based on the architecture of the honey bee AL [44–47] which was able to replicate key features of the AL responses in relation to different odor concentrations and the role of the local GABAergic inhibition. This modelling approach allowed us to estimate the differential distribution of GABA-A and GABA-B receptors-dependent inhibition in iLNs and PNs of the AL network and evaluate gain modulation properties of the AL upon stimulus intensities that vary in the number of recruited glomeruli.

## Results

### GABA-A and GABA-B receptors modulate odor-elicited activity in PNs

**Fig 1:**
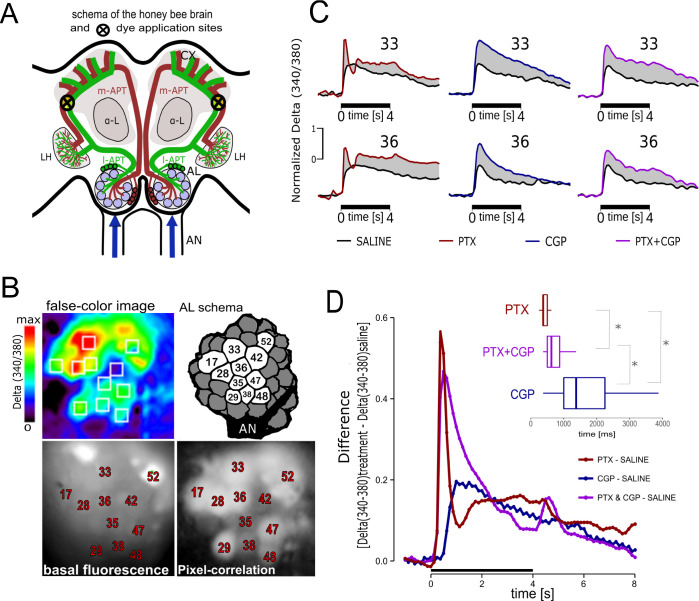
Odor-elicited calcium signals in uPNs and modulation by GABA. **A:** scheme of the honey bee brain and dye injection site. Blue arrows indicate sensory input through the antennal nerves (AN). l-APT (green) lateral Antenno-Protocerebral-Tract, and m-APT (brown) median Antenno-Protocerebral-Tract correspond to uPNs connecting the AL with the mushroom bodies calyces (CX) and the lateral horns (LH). α-L indicates the vertical output lobes of the mushroom bodies that provide visual reference for dye injection. Calcium imaging was performed on glomeruli at the rostro-ventral side of the ALs. **B:** Pseudo-color image showing a representative odor-elicited activity pattern 1 second after odor onset. White squares denote areas integrated as glomeruli. Color-scale represents the Delta of the ratio 340/380 (see methods; imaging analysis). AL schema indicates the glomeruli that were identified in all bees and were used for analysis throughout the study. Glomeruli were identified based on basal fluorescence after FURA-dextran staining (380nm excitation/510nm emission) and aid by neighbors-pixels correlation images (see methods). **C.** Representative calcium imaging traces in identified glomeruli. Numbers on top of each trace indicate glomerulus identification [48] (. Black bars at the bottom of each trace indicate stimulus duration. Odor-elicited calcium signals were measured twice in each animal. The first measurement was obtained under perfusion with physiological saline solution (black traces) and the second measurement was obtained under perfusion of picrotoxin (red), CGP54626 (blue) or a cocktail of both (purple). All traces were normalized by setting to 1 the maximum activity measured for each glomerulus in saline conditions. **D.** Temporal dynamic of GABAergic inhibition. The traces were calculated by subtracting frame-to-frame the trace obtained under perfusion with saline from the trace obtained under perfusion with the blockers for each glomerulus in each animal. The figure shows the average of all glomeruli measured in 9 saline-PTX treated bees; 8 saline-CGP54626 treated bees (blue) and 7 saline-cocktail treated bees. In all traces in which the highest point (peak) of disinhibition caused by the blocker was 0.2 or higher, we recorded the time of the frame in which we measured that peak. Inset: median, 25–75% quartiles, and min/max values of the time to the peak since odor onset. PTX (637 traces) median peak = 375ms (25%-75% = 375-500ms); CGP (275 traces) median peak = 1375ms (25%-75% = 1000-2250ms); PTX+CGP (394 traces) median peak = 675ms (25%-75% = 500-875ms). Kruskal-Wallis test: H (2, N = 1306) = 445,5 p = 0,0001; multiple comparisons: p<0.001 in all cases (*). Note that these times are locked to our acquisition rate, which was one frame every 125 ms. The true peaks of disinhibition may have occurred in the time between frames.

The honey bee AL consists of approximately 160 glomeruli [48,49]. In each glomerulus, approximately 400 ORNs converge onto 5–6 uniglomerular projection neurons (uPNs) [50–52]. A dense network of GABAergic local neurons (LNs) interconnect glomeruli and contribute to synchronize PNs activity and separate odors representations [29,39,46]. Picrotoxin (PTX) and CGP54626 have been used to selectively block GABA-A and GABA-B receptors in the antennal lobe of cockroaches and flies [23,53], as well as in mice olfactory bulb [20,54]. Here we used the same two blockers to study how GABA-A and GABA-B receptors shape responses in projection neurons of the honey bee AL. For this aim we measured odor-elicited activity in the dendritic region of uniglomerular-PNs (uPNs) under physiological saline solution and under perfusion with GABA-A and GABA-B receptors blockers. Uniglomerular-PNs were backfilled with the calcium sensor Fura-dextran [55]. Fig 1A shows a schematic drawing of the honey bee brain indicating uPNs tracts and dye application site. Fig 1B shows different AL visualizations used to aid glomeruli identification. The traces in Fig 1C correspond to representative examples of calcium signals elicited by odor at individual glomeruli. The black traces show signals measured under physiological saline solution and the color traces correspond to the same glomeruli under perfusion with the GABA-A receptor blocker picrotoxin 10uM (red), GABA-B receptor blocker CGP54626 100uM (blue) or a cocktail of both blockers (purple). The shaded area between color and black traces highlight the portion of activity that in control condition is inhibited by GABA through GABA-A, GABA-B or both receptors together. To estimate temporal parameters that describe the contribution of the different GABA receptors, we took the recording performed under a given blocking condition and subtracted from it the recording previously obtained from the same glomerulus but under physiological saline solution. In this way, we obtained for every glomerulus a temporal profile that represents the strength of the GABAergic inhibition on frame-by-frame resolution (8Hz). The different profiles of GABA-A and GABA-B mediated inhibitions were highly consistent across glomeruli and bees (S1 Fig). Fig 1D shows average traces that correspond to the difference between the activity measured under saline and the different blocking conditions. As observed, GABA-A receptors mediate a rapid inhibition that has its highest point between 250 and 500 ms after odor onset. In contrast, GABA-B receptors produce a slower and tonic inhibition with higher strength between 1000 and 2250 ms after odor onset. The inset in Fig 1D shows the median, quartiles, 10% and 90% of the frames in which we measured the highest inhibitions caused by GABA-A and GABA-B receptors. Note that these times are locked to our acquisition rate which was of one frame every 125ms. The distributions where statistically different between treatments (Kruskal Wallis test: H (2, N = 1306) = 445,5 p < 0,0001; multiple comparisons: p<0.001 in all cases (*).

The different temporal contributions of GABA-A and GABA-B receptors are consistent with previous reports in *Drosophila* [23,56] and cockroaches ALs [53]. Interestingly, the interpretation of these profiles must consider that GABA blockers modify PNs activity by reducing inhibition to them, but also by reducing inhibition into iLNs. Thus, when PNs activity is measured in the presence of GABA-A blocker (PTX), the inhibition mediated by GABA-B receptors might be higher than its contribution in normal conditions. Similarly, when activity is measured in the presence of GABA-B blocker (CPG), inhibition mediated by GABA-A receptors might be higher than in normal conditions. In any case, GABA-A and GABA-B receptors shape activity in PNs and LNs both directly and indirectly. The combination of these two effects may explain the rapid decay of the GABA-A dependent inhibition to its lowest point coincident with the highest point of GABA-B mediated inhibition, and similarly, the slow ascending part of the GABA-B mediated inhibition might be explained by early inhibition through GABA-A receptors.

### Distribution of GABAergic inhibition in an AL model. Effect and validation

**Fig 2:**
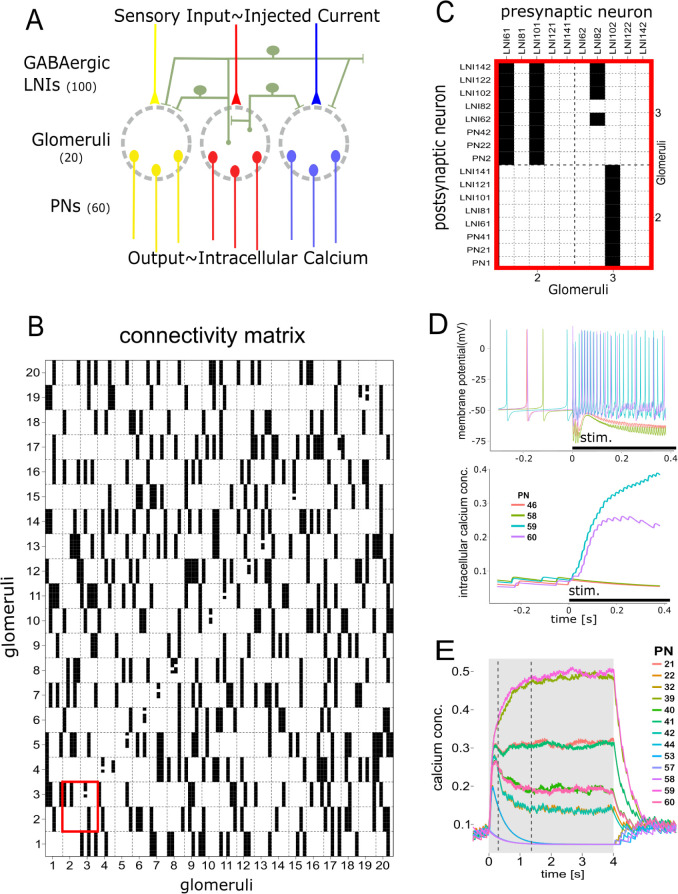
Antennal Lobe model. **A:** Schema of the antennal lobe model. Yellow, red and blue elements indicate input and output in 3 of the 20 glomeruli that constitute the model. Green elements represent local inhibitory neurons that interconnect glomeruli. Local neurons receive excitatory input only in one glomerulus but can inhibit every glomerulus with a probability of 25%. **B.** Example of random generated connectivity matrix. 20 columns represent glomeruli and sub-columns single local neurons. iLNs are clustered in groups of 5 that receive the same depolarizing current that simulates sensory input into one glomerulus. The 20 rows represent the postsynaptic counterparts of the same 20 glomeruli, each one formed by 3 uPNs and 5 iLNs. Black squares indicate that the neuron in the column inhibits the neuron in the row. **C**. Detail of the connectivity between glomeruli 2 and 3. iLNs 61 and 101 receive excitation when glomerulus 2 is excited and inhibit all neurons (LNs and PNs) in glomerulus 3. LNs 81, 121 and 141 receive excitatory input also in glomerulus 2 but do not inhibit glomerulus 2 or 3. iLN 82 receives excitatory input in glomerulus 3 and inhibits all other iLNs in glomerulus 3 but not itself or the uPNs from its own glomerulus. iLN102 receives input in glomerulus 3 and inhibit all neurons in glomerulus 2. LNs 62, 122 and 142 receive excitatory input in glomerulus 3 but do not inhibit glomeruli 2 or 3. **D**. Representative PNs output. Black bars at the abscissa represent the stimulus input. Only four representative uPNs are shown. Upper panel: PNs firing. Lower panel: Intracellular calcium concentration in the same four neurons. **E.** Representative output patterns obtained with intermediate inhibition strength produces heterogeneous responses across uPNs even though the excitatory input is the same square pulse of depolarizing current for all of them.

In the next section we built a mathematical model of the AL that was used to find out the strength and distribution of the GABAergic inhibitions that shape the output pattern of the AL. Even though the precise architecture of the honey bee AL is not yet well known, our model was consistent with previous neuroanatomical and functional descriptions that point out the presence of heterogenous local interneurons that provide patchy and non-uniform lateral inhibition among glomeruli [44–47]. The model network was composed of 20 glomeruli. Each glomerulus housed 3 uniglomerular PNs (uPNs) and 5 inhibitory local neurons (iLNs). Each LN contacted each of the rest of glomeruli with a probability of 25% (Fig 2A). This connectivity generates a network in which there are pairs of glomeruli that are not connected and pairs of glomeruli that are connected with varying strengths, as it has been described for the honey bee AL [46]. PNs acted as post-synaptic elements that integrated excitation from odor stimulation and inhibition from iLNs. In turn, each iLN integrated excitatory and inhibitory inputs from the same two sources and targeted uPNs and iLNs of an average of 5 glomeruli. An example of the connectivity among all neurons in the network is shown in Fig 2B and 2C (see *Methods* for detail: *Network Topology*). Odor stimulation was simulated by injecting a 4-seconds pulse of depolarizing current into uPNs and iLNs that formed the recruited glomeruli. The amount of current and the number of glomeruli that were recruited was varied to simulate different odor intensities. The model output was evaluated based on spike frequency and intracellular calcium concentration (Fig 2D) calculated for each neuron according to the Hodgkin-Huxley model (see *Methods section*: M*odel of Individual Neuron*). An initial inspection of the network´s behavior showed that drastically different outputs could be generated by varying the strength of the GABAaergic inhibitions. When inhibition was high, no activity was elicited at the PNs upon odor stimulation, and when inhibition was low, PNs activity replicated the input stimulus without any evident transformation between input and output patterns. More importantly, we found a wide range of intermediate inhibition strengths, with which the PNs showed a repertoire of different responses even though all of them were stimulated with the same square pulse of depolarizing current (Fig 2E). This result prompted as to further investigate the strength and distribution of GABA-A and GABA-B dependent currents that best mimicked the output of the real AL. For that aim we systemically screened: i) strength of GABA-B dependent currents in PNs, ii) strength of GABA-B dependent currents in iLNs, iii) strength of GABA-A dependent currents in PNs, and iv) strength of GABA-A dependent currents in iLNs. It is important to emphasize that this sort of determinations cannot be obtained by calcium imaging and pharmacological approaches in honey bees.

**Fig 3:**
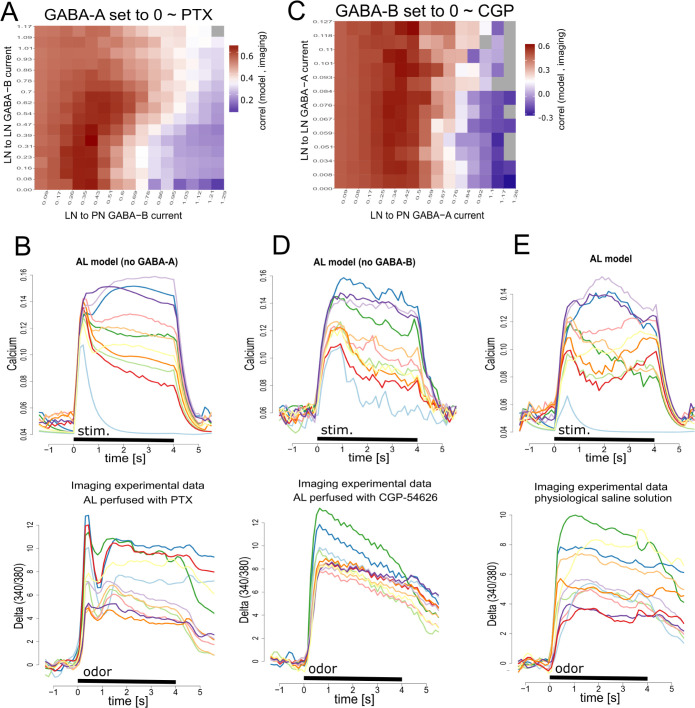
GABA-A and GABA-B dependent currents in iLNs and uPNs. **A.** Search of optimal GABA-B dependent conductance in uPNs and iLNs. Color matrix: similarity index between the AL response obtained by calcium imaging under PTX and the AL model response without GABA-A dependent currents. The strength of GABA-B dependent conductance was varied in iLNs and uPNs to find out conditions that best mimicked the AL output when it was perfused with PTX. Each square represents a new simulation. Values in the X and Y axes indicate the average inhibitory current received by one uPN and one iLN respectively (nA). **B**. Upper panel: AL model response that provided the highest similarity index with the AL imaging response obtained in the presence of PTX (lower panel). **C.** Search of optimal GABA-A dependent conductance. Color matrix: Similarity index between the AL imaging response obtained with CGP54626 and the AL model responses without GABA-B dependent conductance. The strength of GABA-A dependent conductance was varied in iLNs and uPNs to find the conditions that best mimicked the AL output when perfused with CGP54626. **D**. Upper panel: AL model response that provided the highest similarity index with the AL imaging response obtained in the presence of CGP54626 (lower panel). **E.** Upper panel: model AL response patterns obtained combining the optimal conditions determined for GABA-A and GABA-B dependent inhibitions into PNs and iLNs. Lower panel: Imaging AL response to odor in presence of physiological saline solution.

The similarity between the output patterns obtained with the model and the real AL was evaluated in terms of the temporal structure and amplitude of response across glomeruli (see *Methods*: “*Similarity between imaging and model outputs*”). First, we set GABA-A-dependent currents to zero to simulate the experimental condition achieved with PTX (Fig 3B lower panel) and tested different strengths of GABA-B dependent currents in iLNs and uPNs. The matrix in Fig 3A summarizes the search performed to find out the strength and distribution of GABA-B dependent inhibition that best reproduced the calcium traces obtained in the PTX experiments (Eq 7 in *Methods section*: *GABA Synaptic Currents*). Upper and lower panels in Fig 3B show the calcium traces generated by the AL model and the calcium imaging experiments respectively. Second, we set GABA-B dependent currents to zero to simulate the experimental condition achieved with CGP54626 and evaluated the output of the model by testing different strengths of GABA-A dependent currents in iLNs and uPNs (Eq 5 in *Methods section*: *GABA Synaptic Currents*). The matrix in Fig 3C summarizes the similarity indexes calculated between output patterns obtained by the AL model and the pattern obtained in imaging experiments. The Fig 3D upper and lower panels show AL model and imaging outputs obtained without GABA-B receptors. Finally, we set GABA-A and GABA-B dependent conductance to the optimal values determined above and simulated a fully working AL. Interestingly, in spite of its relative simple configuration, the AL model was able to reproduce the main features of the AL responses: an initial abrupt peak rapidly attenuated by action of GABA-A receptors; and slow alternations among glomeruli during the tonic phase of the response, likely due to inhibition mediated by GABA-B receptors (Fig 3E). Remarkably, signal processing in the AL model, decorrelated the activity of PNs, both in intensity and in time, two properties of the AL output that have been linked to optimal separability of odor representations [21,57]. Other type of interactions already described among AL cells were not included in the present model, *i*.*e*. excitatory local interneurons [58], electrical coupling among LNs and PNs [59,60] and pre-synaptic inhibition to the AL [26,56]. Interestingly, the relatively simple version of the AL network was able to reproduce the main features of the real AL responses.

### GABA and gain modulation in the AL

**Fig 4:**
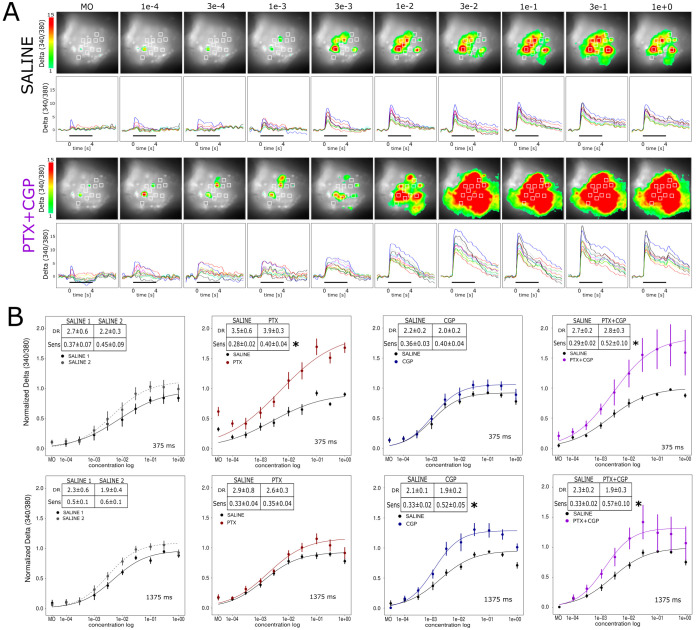
Odor concentration-signal function and GABAergic gain modulation. **A**. Glomerular activity patterns elicited by increasing concentration of 2-octanone. Odor concentrations: head space of 2-octanone pure or diluted in mineral oil: dilutions in liquid phase: 1e+0 (pure); 3e-1; 1e-1; 3e-2; 1e-2; 3e-3; 1e-3; 3e-4 and 1e-4. MO = mineral oil. The head space was further diluted 1/10 in the carrier air stream before reaching the animal. *SALINE*, *upper panel*: false-color images showing Delta (340/380) one second after odor onset in a representative bee. *SALINE*, *lower panel*: Temporal detail of the activity elicited in 11 glomeruli identified in all bees. Black bars in the abscissa indicate stimulus duration. *PTX+CGP*, *upper panel*: false-color images showing Delta (340/380) for the same bee but using both GABA blockers. False-color scale same as in the saline series to evidence the absolute change in activity. *PTX+CGP*, *lower panel*: Temporal detail of the activity elicited in 11 glomeruli. **B.** Four groups of bees were subject to two concentrations series of calcium imaging. From left to right: *saline-saline panel*: group of bees in which the first and second concentrations series were measured under physiological saline solution, N(bees) = 7; *saline-PTX panel*: group of bees in which the first series was under saline and second series under picrotoxin 10 μM, N(bees) = 9; *saline-CGP panel*: group of bees in which the first series was under saline and second series under CGP54626 100 μM, N(bees) = 8; *saline-cocktail panel*: group of bees in which the first series was under saline and second series under the cocktail of picrotoxin 10 μM + CGP54626 100 μM, N(bees) = 7. The analysis was focused on two time windows, each one centered on the imaging frames 375 ms (upper panels) and 1375 ms (lower panels) after odor onset. The figure shows the average and SEM across bees of the Delta (340/380) in the same 11 glomeruli elicited by each odor concentration. Before averaging the bees of the group, the values of Delta (340/380) were normalized in each bee by setting to 1 the highest value obtained for the saline series. After normalization, the relation between odor concentration and Delta (340/380) was adjusted to a logistic function in each bee and for both concentrations series. Insets inside each panel show average and SEM of the Dynamic range (DR) and sensibility (Sens) obtained from the logistic regressions calculated for each bee. * Indicates when the sensibility of the first and the second concentration series were statistically different (Paired T test, p<0.05). Dynamic range was not affected by the GABA blockers.

We evaluated the extent to which GABAergic inhibition contributes to the function that relates odor concentration and output signals in the honey bee AL. For that aim, we measured calcium signals elicited by different odor concentrations under physiological saline solution and under GABA blockers. We used 2-octanone as olfactory stimulus since at mid concentration it activates approximately 50% of the glomeruli at the side of the AL that is accessible for calcium imaging. Fig 4A shows a representative example of the glomerular activation patterns and temporal detail of the calcium traces in identified glomeruli. The example corresponds to a bee that was measured under perfusion with physiological saline solution and afterwards under perfusion with the GABA blockers PTX+CGP. Both concentrations series are represented using the same Delta (340/380) color-scale to clearly show the change caused by the GABA blockers. To compare the effects of the different GABA blockers we averaged the activity from the same indentified glomeruli in all bees and focused the analysis on the imaging frames in which we have previously measured the highest effect of GABA-A and GABA-B dependent inhibitions, i.e. 375 ms and 1375 ms after odor onset (Fig 4B upper and lower panels respectively). The black traces in all panels of the figure show the intensity of the signals elicited upon stimulation with different odor concentrations under perfusion with physiological saline solution. Note that black traces may differ across panels from left to right because each one corresponds to the average of an independent group of bees. As observed, the concentration-response curves describe a sigmoid function that slows down as the odorant reaches higher concentrations. We adjusted the data obtained from each animal and each different treatment to a sigmoid function and calculated dynamic range (DR) and sensibility (Sens) in each case (see methods: Imaging analysis). Fig 4B saline-saline panel shows the group of bees in which the first and the second concentrations series were measured under perfusion with physiological saline solution. No significant change was observed for the DR or Sens between the first and second saline series at any of the two time windows (DR_375ms_: _*paired*_*T-test*: t = 0.98, df = 6, P = 0.36; DR_1375ms_: _*paired*_*T-test*: t = 1.32, df = 6, P = 0.23; Sens_375ms_: _*paired*_*T-test*: t = 1.66, df = 6, P = 0.14; Sens_1375ms_: _*paired*_*T-test*: t = 2.34, df = 6, P = 0.06). In the other three groups of bees, the second concentrations series was measured under saline containing PTX 10μM (saline-PTX panel), CGP54626 100 μM (saline-CGP panel) or a cocktail of both (saline-cocktail panel). The GABA-A blocker PTX increased calcium signals in the time window around 375 ms after odor onset. The analysis of the concentration-response function shows that PTX did not change the dynamic range but changed the sensibility (DR_375ms_: t = 0.73, df = 8, P = 0.48; Sens_375ms_: t = 3.82, df = 8, P = 0.005) which means that PNs responded more abruptly upon changes in odor concentration. This change in sensibility was not as clear during the GABA-B time window (DR_375ms_: t = 0.42, df = 8, P = 0.68; Sens_1375ms_: t = 0.33, df = 8, P = 0.74). Interestingly, a curious observation in this set of bees and not in other, was a drop of activity that took place between no odor and low odor concentrations (Fig 4B, PTX panel). We have not addressed systematically this effect, however it likely corresponds to a higher level of spontaneous activity in the absence of odor, which was exacerbated when GABA-A receptors are blocked. In a third group of bees, the AL was perfused with the GABA-B blocker CGP54626 during the second concentrations series (Fig 4B, saline-CGP panels). Blocking GABA-B receptors increased calcium signals in the time window measured around 1375 ms after odor onset. Blocking GABA-B receptors did not change the dynamic range of the concentration-response function, but it changed the sensibility (DR_375ms_: _*paired*_*T-test*: t = 0.56, df = 7, P = 0.58; DR_1375ms_: _*paired*_*T-test*: t = 2.30, df = 7, P = 0.06; Sens_375ms_: _*paired*_*T-test*: t = 0.98, df = 7, P = 0.35; Sens_1375ms_: _*paired*_*T-test*: t = 3.62, df = 7, P = 0.008). Finally, in the fourth and last group of bees, the second concentrations series was measured with a cocktail of PTX 10μM and CGP54626 100μM. This time a modulation of the gain function was observed in both time windows. The same it happened for the individual blockers, combining PTX and CGP did not change the dynamic range but changed the sensibility of the concentration-response function (DR_375ms_: _*paired*_*T-test*: t = 0.53, df = 6, P = 0.61; DR_1375ms_: _*paired*_*T-test*: t = 2.08, df = 6, P = 0.08; Sens_375ms_: _*paired*_*T-test*: t = 2.70, df = 6, P = 0.03; Sens_1375ms_: _*paired*_*T-test*: t = 2.56, df = 6, P = 0.04).

### Gain modulation and odor concentration invariance

**Fig 5:**
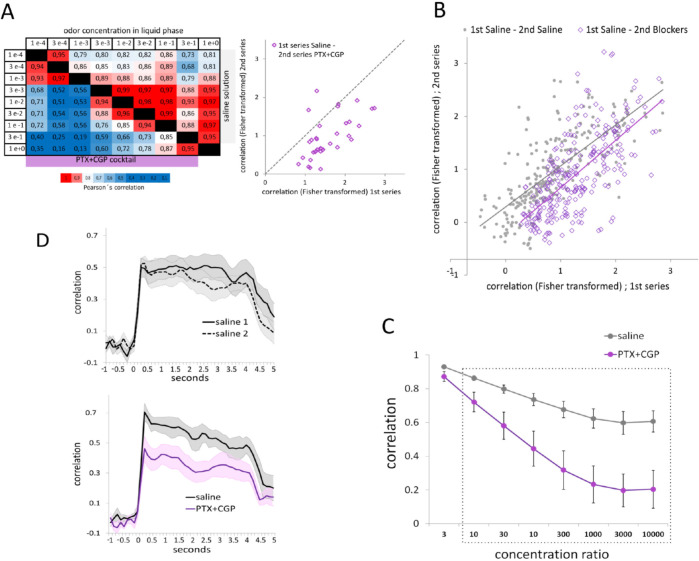
GABA and stability of the glomerular code. **A**. Left: Cross-concentrations correlation matrix in a representative bee. Upper half matrix: Pearson´s correlations among activity patterns elicited by different concentrations during the saline series. Lower half matrix: Pearson´s correlation values among activity patterns elicited by different concentrations in the same bee but during the GABA blockers series. Color scale: red for highest and blue for lowest correlation values. Right panel: Same data as in the correlation matrix, but values were Fisher z-transformed. Each square corresponds to a pair of activity patterns elicited by two different concentrations located in the scatterplot, according to their correlation in the first series (abscissa) and their correlation in the second series (ordinate). **B**. Fisher z-transformed correlations obtained for all pairs of activity patterns during the first and the second concentrations series for all bees that underwent two series under physiological saline solution (gray dots) and all bees that underwent the first series under saline and the second series under GABA blockers cocktail (purple). Lines correspond to the respective trendlines. **C.** Mean and SEM of the correlation values among pairs of activity patterns elicited by different concentrations pooled according to the concentrations ratio. Values in the abscissa correspond to the concentration ratio between the activity patterns that were compared, i.e. 1e-2 vs. 1e-4 and 3e-1 vs. 3e-3 are a ratio of 100 in both cases. Gray line: concentrations series under physiological saline solution; purple line: concentrations series under GABA blockers cocktail. The box indicates the cases in which the correlation values under saline and under the blockers were statistically different. Paired t-test, N = 7 and p<0.05. **D**. Correlation values calculated frame by frame between pairs of activity patterns during the first or during the second concentration series. Correlation values among activity patterns elicited by different odor concentrations (all concentration ratios of 10 or higher were pooled). In all cases: Bold lines indicate average and shaded areas denote SEM of 7 bees. Upper panel: group of bees with two series under saline, N(bees) = 7. Lower panel: group of bees with first series saline and second series GABA blockers cocktail N(bees) = 7 (lower panel).

Uniglomerular-PNs connect the AL with the Kenyon cells in the calyces of the mushroom bodies (MBs) (Fig 1A) [61–63]. At the mushroom bodies, odors are encoded by small subsets of Kenyon cells that are tuned to respond upon activation of specific ensembles of projection neurons [64–67]. Changes in the combination of PNs produce the activation of different Kenyon cells [66] which may produce the perception of different odors. Therefore, to keep odor perception stable across a certain concentrations range, activation patterns at the level of PNs must be able to attenuate concentration differences [17]. In the present study we were able to identify the same set of glomeruli in all bees and this allowed us to analyze the stability of the pattern that encodes the odor across concentrations and across treatments. The pattern stability was analyzed based on Pearson´s correlation coefficients between glomerular patterns elicited by different odorant concentrations. The color-coded table in Fig 5A corresponds to a representative bee and contains the correlation values obtained for pairs of activity patterns elicited by all different concentrations. Above the diagonal are the correlation values calculated for pairs of activity patterns obtained under perfusion with the physiological saline solution and bellow the diagonal are the correlation values obtained among de same pairs of patterns but under PTX+CGP (9 odor concentrations make 36 possible comparisons). The left panel in the Fig 5A shows the same 36 comparisons, but graphically arranged to show that activity patterns elicited by different concentrations were more similar during the first concentration series performed under saline (abscissa), than during the second series performed under the GABA blockers cocktail (ordinate). Note that for this plot and the one in Fig 5B, the Pearson’s correlation values were Fisher z-transformed to obtain higher resolution of correlation values close to 1. The observed result was further confirmed by performing a principal component analysis that used temporal and spatial information of the activation patterns (S2 Fig). Fig 5B extends the correlation analysis among activity patterns elicited by different odor concentrations to all animal in the group of bees that underwent two concentrations series under saline solution (7 bees x 36 comparisons; gray dots in the Fig 5B) and all animals that were recorded first under saline and second under the blockers cocktail (7 bees x 36 comparisons: purple squares in the Fig 5B). Each point in the figure corresponds to one pair of odor concentrations, located in the scatter plot according to the correlation coefficients among the patterns measured during the first concentrations series (x-axis) and during the second concentrations series (y-axis). As observed, a large proportion of pairs in the saline-blockers group (purple points) are skewed towards lower correlation values in the second series (blockers: ordinate) than in the first series (saline: abscissa).

Next, we asked whether the comparison between distant concentrations were more affected by blockers than the comparison between closer concentrations. For that aim, we pooled and averaged correlation values according to the relative distance between the two concentrations that are compared (for example, 1e-01 vs. 1e-02 and 3e-01 vs. 3e-02, are both considered comparisons among concentrations ratio = 10). Fig 5C shows the correlations pooled and organized according to the ratio between concentrations that are compared in the group of bees that were measured first under saline (gray markers and line) and later under PTX+CGP (purple markers and line). As observed, GABA blockers reduced the correlation coefficients between activity patterns elicited by concentrations that are separated by a ratio of 10 or higher. The ratios inside the box are the cases in which the difference between saline and PTX+CGP was statistically significant (*ratio3*: t = 2.20, df = 6, P = 0.07; *ratio10*: t = 3.06, df = 6; P = 0.02; *ratio30*: t = 2.96, df = 6, P = 0.02; *ratio100*: t = 3.37, df = 6, P = 0.01; *ratio300*: t = 3.66, df = 6, P = 0.01; *ratio1000*: t = 3.55, df = 6, P = 0.01; *ratio3000*: t = 4.03, df = 6, P = 0.01; *ratio10000*: t = 2.90, df = 6,P = 0.02). Finally, we extended the analysis to every 125ms acquisition frame, from one second before odor onset until one second after odor offset (Fig 5D). All correlation coefficients between patterns elicited by concentrations that are separated by a ratio of 10 or larger were averaged. As observed, 375ms after odor onset, the correlation reaches its maximum value. The upper panel shows the results obtained in bees that underwent both series under the physiological saline solution. The lower panel corresponds to bees that underwent the second series under GABA-blockers. As shown in the figure, the cross-concentrations correlation is affected throughout the whole stimulus period.

### Gain modulation increases with the number of active glomeruli

**Fig 6:**
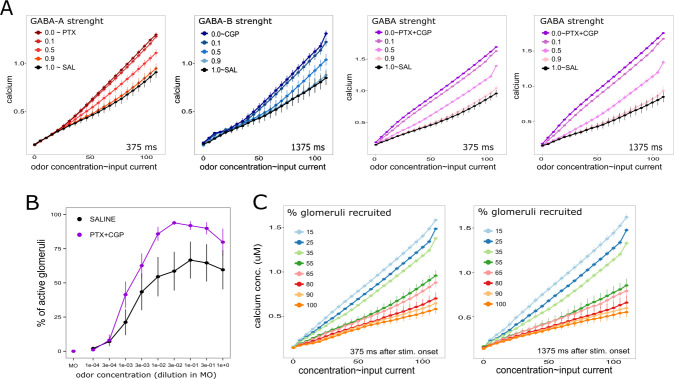
GABA and gain modulation in the AL model. **A.** Antennal lobe model output for increasing input currents. Values indicate mean and SEM of the intracellular calcium concentration in active glomeruli (uM). Recruited glomeruli received the same amount of depolarizing current which varied from 0 to 100 nA. The black series correspond to optimal GABA-A and GABA-B dependent conductance obtained in the previous section and therefore are considered as a “saline” experimental condition. The red graded series corresponds to gradual reduction of the GABA-A dependent conductance, expressed as a proportion of the highest strength. The blue graded series corresponds to gradual reduction of the GABA-B dependent conductance measured 1375 ms after stimulus onset. The purple graded series correspond to the gradual reduction of both, GABA-A and GABA-B dependent conductance measured 375 and 1375 ms after odor onset. B. Mean and SEM of the percentage of glomeruli activated by different odor concentrations. The data corresponds to the group of bees that were first treated with physiological saline solution (black) and second with the GABA-R blockers cocktail (purple) N(bees) = 7. **C.** AL model output as function of the injected current and the number of glomeruli that are recruited. The data represents mean and SEM of the intracellular calcium determined in active projection neurons, 375 and 1375ms after stimulus onset.

We tested the response of the AL model to different stimulus intensities and evaluated in it the role of GABAergic inhibition. Since we observed in the previous experimental section that blocking GABA-A and GABA-B receptors did not affect the dynamic range of the function, we only tested changes in the sensibility of the function that relates stimulus intensity and calcium signals. The stimulus strength was initially controlled by the amount of injected current and fixing the percentage of glomeruli that were recruited to 55%. First, we tested the model in conditions that would correspond to the experiments with PTX. For that aim, the conductance associated to GABA-B receptors in uPNs and iLNs was set to the optimal values determined above and the conductance associated to GABA-A receptors was progressively reduced in successive trials starting from its optimal value (black line in Fig 6A) to eventually reach zero (orange-red-brown lines). The response intensity upon different input strength was calculated by averaging the PNs internal calcium concentration 375 ms after stimulus onset. As observed, the effect of removing GABA-A dependent inhibition emerges progressively as the stimulus intensity increases. Second, we set the conductance associated to GABA-A receptors to the optimal value previously determined and the conductance associated to GABA-B was gradually reduced starting from the optimal value (black line) to finally reach zero (blue graded lines). This time, the analysis was focused on the 1375ms window. As shown in Fig 6 as the strength of GABA-B dependent inhibition is reduced, the slope of the signal/stimulus function increases. In comparison to GABA-A, blocking GABA-B produced slight changes that, although small they were evident already from very low stimulus intensities. Third, we tested the behavior of the AL model by simultaneously reducing both, GABA-A and GABA-B dependent currents (Fig 6 purple lines). This time, the contribution of GABA was evident for all stimulus intensities. The observation that GABA-A or GABA-B receptors were sufficient to provide gain control at low stimulus intensities when they were alone might be explained by the fact that iLNs also inhibit other iLNs. Thus, when GABA-A is blocked, iLNs receive less inhibition and GABA-B mediated inhibition into PNs is enhanced. Similarly, when GABA-B is blocked, iLNs receive less inhibition and GABA-A mediated inhibition into PNs is enhanced.

The previous situation in which we increase the intensity of the stimuli by increasing the input currents without changing the number of glomeruli that are recruited seems to be far from real. Therefore, in this last section we tested the behavior of the AL model when both input parameters change; the input current and the number of glomeruli. First, we came back to the calcium imaging experiments and evaluated how the number of glomeruli that are recruited changes when the odor concentration increases. We counted glomeruli as being activated by the odor when their activity upon odor onset was two times higher than the SD of the baseline activity measured before odor onset. The number of glomeruli that are recruited by the odor increases with odor concentration and reaches a maximal value of about 60% of the glomeruli (Fig 6B). Instead, almost 100% of glomeruli were active when GABA receptors were blocked, which means that without GABAergic inhibition, no glomerular code would be possible at higher odor concentrations. Furthermore, the observation that activity can reach 100% of the glomeruli indicates that the maximum value of 60% obtained in control condition was not due to a limit of the imaging technique or responsiveness of the projection neurons. Interestingly, it indicates that even saturating input levels can be handled by the GABAergic network to keep odor representation within a range of activity that is compatible with the combinatorial code.

Finally, the AL model gave us the opportunity to evaluate the relation between the number of glomeruli that are recruited and the power of the GABAergic network that keeps odor elicited activity within a range that makes possible the glomerular code. We tested the behavior of the AL network varying the amount of current and the number of glomeruli into which this current was injected. Fig 6C shows the average response of the PNs as a function of the current injected and the percentage of glomeruli that are recruited. When the proportion of glomeruli that are recruited increases, the slope of the gain function decreases. Interestingly, this is an experiment that is only possible using the model, since independently manipulating the intensity of the stimulus and the number of recruited glomeruli cannot be achieved in physiology experiments in most experimental models. In summary, the results indicate that the power of the gain control function is proportional to the number of glomeruli that are recruited and depends on GABA.

## Discussion

We have described the contribution that signal-processing in the AL does to reduce the effect of odor concentration in the representation of odor identity. The analysis was focused on the role that GABA-A and GABA-B receptors play in terms of the amplitude and temporal profiles of the signals that convey odor information from the ALs to the mushroom bodies and lateral horns of the honey bee brain. We found that GABAergic inhibition increases the similarity between activity patterns elicited by different concentrations of the same odor. Using a computational model of the AL we were able to extend our characterization of the AL network beyond experimental possibilities. Despite simple connectivity rules and cell-to-cell interactions mediated solely by GABAergic inhibitions, the model was efficient in reproducing key features of AL responses in relation to odor representation across the intensities. Interestingly, the model provides a simple computational solution that is of interest for the design of bioinspired systems used in object recognition technologies.

### GABAergic modulation of the odor concentration-signal function

The relation between odor concentration and PNs activity was described by focusing on the number of glomeruli that are recruited (Fig 6B) and the intensity of calcium signals (Fig 4B). In control conditions, the number of glomeruli that were recruited increased with odor concentration and did not surpass a maximum of 60% of the glomeruli. In contrast, when GABA receptors were blocked, the relation was much steeper and 100% of the scrutinized glomeruli were active when stimulating with the five highest concentrations. The AL model gave us the possibility to study the relation between the number of glomeruli that are recruited and the strength of the gain modulation function (Fig 6C). Interestingly, the slope of the input-signal function scales down with the number of recruited glomeruli: the more glomeruli are recruited, the less the output signals are affected by the increase in input currents. This result must be interpreted in the context of real stimuli; low odor concentrations recruit only few glomeruli and gain control must play no, or a minimal role, as long a no additional glomeruli are recruited. Instead, higher odor concentrations recruit more glomeruli, and the inhibitory gain control is scaled accordingly. This observation is consistent with studies in *Drosophila* which demonstrated that the activity at single projection neurons scales in inverse proportion with the number of co-activated receptors [16].

### Glomerular odor representations

Kenyon cells, the post-synaptic partners of the uPNs in the mushroom bodies, behave as decoders that detect specific ensembles of co-activated projection neurons [65,67–69]. In this context, it is understood that odor recognition requires stable PNs ensembles, rather than globally stable activity levels [70]. The identification of the same glomeruli across animals allowed us to focus the analysis on the stability of the glomerular pattern that encodes the odor (Fig 5). Blocking GABA receptors severely affected the correlation among glomerular patterns elicited by different concentrations of the same odor (Figs 5C and S2). Interestingly, a previous study in honey bees, showed that increasing odor concentration increases the separation among patterns of activity elicited by different odors, and suggested that generalization across different concentrations of the same odors is possible thanks to smooth transitions among the patterns elicited by different concentrations [17,55,71]. Our results suggest that local computations based on the GABAergic network contribute to both, discrimination between odors and generalization between concentrations. In the first case, increasing odor concentration broadens the pattern of receptors that are recruited [7]. This broadening produces pattern overlap between odors and potential loss of discrimination. GABAergic gain modulation in the AL would sharpen odor representation limiting activity to a core set of glomeruli. Accordingly, it was shown in honey bees that different odors are better discriminated at high than low concentrations [72]. In the second case, the pattern of activity elicited by high odor concentration includes the glomeruli activated by low concentrations, plus secondary glomeruli that might alter the perceptual quality of the odor. As GABAergic modulation is stronger in higher than in lower odor concentrations, gain modulation brings the pattern elicited by the higher concentrations closer to the one elicited by the lower concentrations, thus improving generalization, even though the input maps can change markedly [19].

### AL model architecture

Novel computational approaches aimed at understanding how the AL network integrates and process information from ecologically relevant stimuli should incorporate current knowledge about the intra and interglomerular organization of the AL circuit [73,74],. In our study, glomeruli were functionally defined by the fact that iLNs and PNs stemming from the same glomerulus receive the same excitatory and inhibitory input, and second by the constraint that LNs do not inhibit PNs from its own glomerulus. The consequence is a highly interconnected network in which the probability that a given glomerulus does not receive inhibition from another given glomerulus was as low as 0.23 and the number of iLNs received from another glomerulus could vary between 0 and 5 which provided heterogeneous distribution of the inhibitory inputs [46,75]. LNs inhibited PNs but could also disinhibit them by inhibiting other iLNs [76,77]. Thus, the total amount of inhibition received by any PN in each glomerulus was directly related with the number of glomeruli that were recruited and inversely related with the excitatory input that is received, since iLNs stemming from the same glomerulus receive the same excitatory input as the PNs and could inhibit incoming iLNs. The absence of self-recurrent inhibitory feedback makes that only activation patterns that include two or more glomeruli are subject of gain modulation. Inhibitory feedback is based on the existence of odors that elicit multi-glomerular patterns and depends on reciprocal inhibition among co-activated glomeruli. Indeed, this design is consistent with the way in which general odors are detected and encoded [7]. Accordingly, it was shown in larvae of *Drosophila melanogaster*, that GABAergic neurons that provide gain control are recruited only upon summed activation of at least two independent olfactory channels, but not when the sensory input is restricted to one channel [14]. In this context, pheromones that are detected and encoded by specific labeled lines [78–81] would benefit from having no recurrent inhibition. There are however several pheromones that are encoded by combinations of co-activated channels [82,83]. Those patterns should not remain subordinated to concurrent odors. Thus, it is expected that iLNs network should incorporate hierarchical processing of the olfactory information [84].

### Distributed gain control

Gain control in the antennal lobe or the olfactory bulb is not absolute [16,19,25,55,71,85]. The transformation that takes place in the AL attenuates the effect of the concentration but does not make it independent (Fig 4). In a way, information about concentration should not get lost, since different concentrations of the same odor may have different or even opposite values [86]. A mechanism to separately encode quality and quantity has been proposed for honey bees and other hymenoptera. These species possess two parallel pathways of uPNs. The lateral antenno-protocerebral tract (l-APT) that we have measured in the present study, and the medial antenno-protocerebral tract (m-APT). Both tract were proposed to encode identity and quantity respectively [35,62,82]. Furthermore, activity at the synapses between uPNs of the lACT and Kenyon Cells were shown to encode odor identity in a concentration invariant way [87]. In this region, uPNs boutons receive GABAergic inhibitions derived from local interactions [67,88] as well as GABAergic feedback from the mushroom body output lobes [89,90]. Thus, the partial gain modulation reported in the AL must be considered as part of a progressive sequence of transformations along the olfactory pathway [1,15].

### Gain modulation and plasticity

As observed, the function that relates odor concentration and its internal representation depends on a finely balanced strength of inhibitory synapses in the AL. Interestingly, previous studies showed that changes in iLNs of the AL contribute to associative and non-associative plasticity linked to the ability to ignore or detect odors according to their value [41–42,91–94]. Now, it can be expected that those changes might also have a consequence on the gain function, likely improving the intensity-invariant representation of learned odors. In this way, two critical functions of the AL network, i.e. contributing to a concentration-invariant representation of odors and detection of relevant odors, might arise at the same time and based on the same mechanisms, providing animals the ability to detect and recognize meaningful odors presented at varying concentrations.

## Materials and methods

### Animals

Honey bee *Apis mellifera* pollen foragers were collected at the entrance of regular hives located at the campus of the University of Buenos Aires (34° 32’ S; 58° 6’ W). The bees were briefly cooled and restrained in individual holders suited for brain imaging recording [33]. After recovery from cooling, bees were fed 5 μL of a 1.0 M sucrose solution and remained undistributed until feeding *ad libitum* in the evening. At all times bees were kept in a humid box at room temperature (20–24°C) on a 12:12 h light:dark cycle. All experiments started 1 day after capture.

### Odor stimulation

The odorant used was 2-octanone (TCI America, Portland OR). The odor delivery device, from now on the “odor-gun”, was designed to provide 9 concentrations of 2-octanone or clean air upon demand. The odor-gun consisted of ten independent channels, each of them attached to 6 ml glass vial that contained the odorant diluted at different concentrations. Each vial had a liquid volume of 300 μl. The dilutions in the liquid phase were in mineral oil (V/V): 1e-04, 3e-04, 1e-03, 3e-03, 1e-02, 3e-02, 1e-01, 3e-01 and pure (1e+01) 2-octanone. The tenth vial contained only mineral oil (MO). The saturated headspace inside the vials was used for stimulation. The odor-gun had a central charcoal filtered air stream of 500 ml/min in which the headspace of the vials was pushed by nitrogen at a flow of 50 ml/min (nitrogen avoids odorant oxidation). Odor stimulation lasted 4 seconds, which makes that 3,3 ml of the headspace of the vial were delivered to the bee. The final odor concentration that was delivered to the bee resulted 1/10 of the concentration in the headspace of the vial. Opening and closing of the odor channels were controlled by solenoid valves (LFAA1200118H; The LEE Company) synchronized by the imaging acquisition software (TillVision). During periods without odor stimulation the charcoal-filtered air stream continuously ventilated the antennae. A gentle exhaust located 10 cm behind the bee removed the odors.) Odor stimulation was always separated by 1-minute intervals.

### Projection neurons staining

The head of the bee was fixed to the stage with soft dental wax (Kerr Sybron Dental Specialties, USA). A window was cut in the head capsule between the joints of the antennae and the medial ocellus. The glands were carefully moved aside until the mushroom body α-lobes were visible which serve as spatial reference for staining. PNs were stained by backfilling with the calcium sensor dye Fura-dextran (potassium salt, 10,000 MW; Thermo Fisher Scientific, USA). The tip of a glass microelectrode coated with dye was inserted into both sides of the protocerebrum, dorsolateral to the α-lobes where the antenna-protocerebral tracts enter the lateral calyces of the mushroom bodies [63] (see Fig 1A first panel). The dye bolus dissolved into the tissue in 3–5 seconds. The window was closed using the piece of cuticle that had been previously removed and it was sealed with eicosane (Sigma-Aldrich) using a low temperature soldering needle. Twenty minutes after staining, the bees were fed with 1 M sucrose solution and left undisturbed until the next day. Before imaging, the antennae were fixed pointing towards the front using eicosane. The head capsule was opened, and the brain was rinsed with Ringer solution (in mM: NaCl, 130; KCl, 6; MgCl_2_, 4; CaCl_2_, 5; sucrose, 160; glucose, 25; and HEPES, 10; pH6.7, 500 mOsmol; all chemicals from Sigma-Aldrich). The glands and trachea covering the ALs were removed. Only ALs that presented homogeneous staining of all visually accessible glomeruli were used for imaging. Body movements were prevented by gently compressing the abdomen and thorax with a piece of foam. A second hole in the head capsule was cut between the antennae and the mandibles, and the compact structure of muscles, esophagus and supporting chitin was lifted and put under slight tension. After preparation, the bees were placed in the microscope and were let to recover for 20 minutes before starting the imaging experiments.

### Calcium imaging

Calcium imaging was done using a EMCCD iXon camera (ANDOR, Belfast, UK) mounted on an upright fluorescence microscope (Olympus BX-50WI, Japan) equipped with a 20× dip objective, NA 0.95 (Olympus). Filter- and mirror-set: 505 DRLPXR dichroic mirror and 515 nm LP filter (Till-Photonics, Gräfelfing, Germany). Excitation light was provided by a Polychrome V (Till-Photonics) which alternated between 340 and 380 nm. Acquisition protocols were made using the software TillVision (Till-Photonics). The sampling rate was 8 Hz, and the spatial resolution was 125×125 pixels binned on a chip of 1000×1000 pixels. The intensity of the fluorescence lamp was controlled from the imaging acquisition software to get exposure times of 20 ms and 5 ms for 340 and 380 nm respectively. Each imaging session consisted of 20 measurements of odor-elicited activity (2 trials for each odor concentration). The odor concentrations were presented in increasing order and trials were separated by 1 min intervals. Two complete series of increasing odor concentrations were performed for each bee. Series were separated by a 10 minute interval giving time to change the perfusion solution between series. Each measurement lasted 10 seconds and the odor presentation lasted 4 seconds, from seconds 2 to 6. The first concentration series was always performed under the physiological saline solution. The second concentration series depended on the experiment: physiological saline solution, picrotoxin (Sigma), CGP54626 (Sigma) or cocktail of both blockers in all cases prepared in the same saline solution.

### Pharmacology

The bees were divided into four groups, each treated with different solutions during the second odor concentration series: control bees (with physiological saline solution), PTX (picrotoxin GABA-A non-competitive antagonist) 10 μM [15], CGP54626 (GABA-B antagonist) 100 μM [23,53] or a cocktail of both (PTX+CGP) 10 μM + 100 μM respectively.

### Imaging analysis

Imaging analysis was done using software written in IDL (Research Systems, CO, USA) by Giovanni Galizia (University Konstanz, Germany) and in R by Emiliano Marachlian. Each measurement consisted of two sequences of 80 fluorescence images each, obtained by alternating 340 and 380 nm excitation light conveniently used for ratiometric determination of changes in intracellular calcium concentration (*Fi*_340_, *Fi*_380_, where *i* is the number of the image from 1 to 80) [12]. For each pair of image *Fi*, we calculated pixel-wise the ratio *Ri* = (*Fi*_340 nm_ / *Fi*_380 nm_) × 100 and subtracted the background *Rb*, obtained by averaging the *Ri* values 1 second before odor onset [*Rb* = 1/8 (*R*_*8*_ *+ … + R*_*16*_)]. The resulting values indicated as “Delta (340/380)” in the figures, represent the change of fluorescence from the reference window and are proportional to the changes in the intracellular calcium concentration. The analysis of odor-induced activation patterns in the present study was based on signals from 11 glomeruli that were identified in all bees on the basis of their morphology and position using the published atlas of the honey bee AL [44,48]. Glomeruli are visible in the raw fluorescence images at 380 nm excitation light after backfilling the PNs with FURA (Fig 1B). In addition, we used a tool written in IDL by Mathias Ditzen (Freie Universitaet Berlin, Germany) that segments the image based on the degree of correlated activity between neighboring pixels. Pixels stemming from the same glomerulus are highly correlated. This tool provides images in which glomeruli are discrete units separated by dark boundaries (Fig 1B) and helps their identification. The glomerular activation was calculated by averaging activity in a square area of 7×7 pixels that correspond to 23×23 *μ*m and fits within the size of the glomeruli. To calculate the function that relates odor concentration and intensity of the calcium signals, we averaged the Delta (340/380) determined for the eleven identified glomeruli in each animal and each treatment during the indicated frames, and adjusted them to a logistic function using nonlinear least squares in R:

$$Delta\;(340/380)=\frac{a}{1+e^{-b\;conc\;c}}$$

Based on the obtained a, b and c we calculated Dynamic Range (DR) and sensibility (sens):

$$DR=EC\;90-EC\;10=\frac{\log\;(\frac{1}{9})+c}{-b}+\frac{\log\;(9)+c}{b}$$

$$Sens=\frac{0.8\;a}{DR}$$

DR and sens was obtained for each animal and each concentration series. Statistical analysis was based on Paired T test of DR and sens obtained in the first series vs. the second series.

### Antennal lobe model

#### Network topology

Since we know the general architecture of the honey bee antennal lobe [47,95] but not the absolute connectivity of each individual neuron, we opted to establish standard connectivity rules between neurons and glomeruli that vary within a range of possibilities that were randomly assigned. The AL model contained 20 glomeruli. Each glomerulus had 3 PNs and 5 iLNs (Fig 2A). Each LN received excitatory input in one glomerulus and could inhibit every glomerulus with a probability of 25%. This degree of connectivity generates a network in which there are pairs of glomeruli that are not connected and pairs that are connected with variable strength, similar to the AL network described for honey [46,96]. When a given iLN inhibited one glomerulus, it inhibited all neurons in it (3 PNs and 5 iLNs), except when it was in the glomerulus in which this iLN received excitatory input (home-glomerulus) in which case it inhibited the other 4 iLNs but not the 3 PNs or itself. Thus, every LN inhibited an average of 5 glomeruli and every glomerulus received inhibition from an average of 25 LNs. An example of the connectivity among the 160 neurons in the network is shown in Fig 2B. Column and row numbers indicate glomeruli. As example, the intersection between glomeruli 2 and 3 is enlarged for visualization of the connectivity between the neurons in these two glomeruli (Fig 2C). A black square in the intersection indicates that that the neuron in the column makes synaptic contact with the neuron in the row. The neurons in the columns represent the pre-synaptic partners and the neurons in the rows are the post-synaptic ones. The columns contain only iLN because the PN did not act as pre-synaptic partners in the network. When two neurons were connected, it was considered that both, GABA-A and GABA-B receptors participated of the inhibition.

### Odor stimulation

Odor stimulation was simulated by injecting a pulse of depolarizing current into the 3 uPNs and the 5 iLNs that formed the recruited glomerulus. The active glomeruli were selected based on a random function. Excitatory input, as well as the probability of the glomeruli to receive excitatory input, was varied to change stimulus intensity. All neurons (uPNs and iLNs) in one glomerulus received the same excitatory input. All glomeruli that were recruited by the odor (and therefore all neurons inside it) received the same amount of depolarizing current. Membrane potential and ionic currents were calculated for every neuron following the Hodgkin and Huxley model [97] adjusted by the parameters in Table 1. The excitatory input that simulated odor stimulation consisted in a depolarizing current that lasted 4 seconds with the form:

$$I=I_{0}\;sc\;e^{\frac{rate(t-t_{0})}{1000}}$$

where I_0_ constitutes the parameter that was changed to vary the size of the sensory input, *sc* is the connection strength and *rate* corresponds to sensory adaptation (values in Table 1). The input *I*_*o*_ was varied across simulations between 0 and 100 nA to represent different odor concentrations.

**Table 1:** The table summarizes the values of all the parameters included in the model.

Parameter	Symbol	iLNs	PNs
Soma capacity	*C*_*s*_	10.0 nF	10.0 nF
Axon capacity	*C*_*ax*_	10.0 nF	10.0 nF
Resting potential	*V*_*L*_	-45 mV	-45 mV
Leak conductance	g_l_	0.16 *μ*S	0.16 *μ*S
Couple axon-soma conductance	g_AS_	10.0 *μ*S	65.0 *μ*S
I_Ca_powers values Ca and rest potential	*M*_*Ca*_ *H*_*Ca*_ *V*_*rCa*_	3 0 0 mV	3 0 0 mV
I_A_powers values A and rest potential	*M*_*A*_ *H*_*A*_ *V*_*A*_	1 0 -60 mV	1 0 -60 mV
I_Na_powers values Na and rest potential	*M*_*Na*_ *H*_*Na*_ *V*_*Na*_	2 1 50 mV	2 1 50 mV
I_Kd_powers values Kd and rest potential	*M*_*Kd*_ *H*_*Kd*_ *V*_*Kd*_	1 0 -60 mV	1 0 -60 mV
I_KCa_powers values KCa and rest potential	*M*_*KCa*_ *H*_*KCa*_ *V*_*KCa*_	1 0 -60 mV	1 0 -60 mV
Threshold potential	V_t_	-51.7 mV	-52.1 mV
Calcium dynamic dissipation	MU	1.5	1.6
Strength connecting	*Sc*	0.5	0.7
Sensory adaptation	*rate*	0.05	0.05
GABA rest potential	V_GABA_	-90 mV	-90 mV
	K_fGABA-A_	10.0 ms^-1^	
	K_rGABA-A_	0.1 ms^-1^	
	K_dGABA-B_	100	
	K_1GABA-B_	0.6 mM^-1^ms^-1^	
	K_2GABA-B_	0.001	
	K_3GABA-B_	0.3	
	K_4GABA-B_	0.0025	

### Model of individual neurons

Each PN and LN was modeled as two compartments (soma and axon) that included voltage and Ca^2+^ dependent currents based on the Hodgkin-Huxley model [97]. Membrane potential of PNs and LNs was modeled by the two differential equations for the soma and axon respectively (1). Both contained leak and axon-soma coupled currents. The membrane potential equation for the soma included the calcium current *I*_*ca*_, the transient potassium current *I*_*A*_, synaptic GABA-A and GABA-B current *I*_*syn*_, a current that represented noise *I*_*r*_ and the sensory input current *I*. The membrane potential equation for the axon included the sodium current *I*_*Na*_, the rectifier potassium current *I*_*kd*_ and the potassium dependent calcium current *I*_*KCa*_.


$$C_{s}\left(\frac{\partial V_{s}}{\partial t}\right)=g_{1}(V_{s}-V_{L})-g_{AS}(V_{s}-V_{a})-I_{Ca}-I_{A}-I_{syn}+I_{r}+I$$

$$C_{ax}\left(\frac{\partial V_{a}}{\partial t}\right)=g_{1}(V_{a}-V_{L})-g_{AS}(V_{a}-V_{s})-I_{Na}-I_{Kd}-I_{KCa}$$ (1)

*C*_*s*_ and *C*_*ax*_ are the soma and axon capacity constants respectively (all parameters are showed in Table 1). *V*_*s*_ and *V*_*a*_ are the membrane potential of soma and axon membrane respectively. The two first terms to the right of the equation are lineal. The conductances g_l_ and g_AS_ are constants. The first one corresponds to leak currents, and the second one is the axon-soma coupling term. The *I*_*ca*_, *I*_*A*_, *I*_*Na*_, *I*_*Kd*_ and *I*_*KCa*_ are described by the general Eq (2).


$$I_{Ion}=g_{Ion}\;m_{Ion}^{M_{Ion}}\;h_{Ion}^{H_{Ion}}\;\left(V_{\frac{s}{a}}-V_{rIon}\right)\;f_{Ion}$$ (2)

The subindex *Ion* indicates that this parameter or variable is ion specific. The gating variables *m* and *h* are temporal variables each one with its own dynamic Eq (3). Each ionic current has its own gating variables and in the Eq (2) are raised to different powers *M* and *H*. *V*_*s/a*_ indicate the adequate soma or axon membrane potentials respectively *(V*_*s*_ for *I*_*C*a_ and *I*_*A*_ and *V*_*a*_ for *I*_*Na*_,*I*_*Kd*_ and *I*_*KCa*_) and *V*_*rIon*_ is the rest potential for specific ion (Table 1). *f*_*Ion*_ is 1 in all currents except in *I*_*Ca*_ in which

$$f_{Ca}=\frac{1}{1+e^{\frac{2V_{s}}{24.4}}}$$

$$\frac{\partial m_{Ca}}{\partial t}=0.1\left(\frac{1}{1+e^{\frac{-V_{s}-39.1}{2}}-m_{Ca}}\right)$$

$$\frac{\partial m_{A}}{\partial t}=\frac{1}{\left(1+e^{\frac{10}{8}V_{s}}-m_{A})(350-349(1+e^{V_{s}+\frac{46}{4}})\right)}$$

$$\frac{\partial m_{Na}}{\partial t}=\frac{0.32(V_{t}-V_{a}+18)}{e^{\frac{V_{t}-V_{a}+18}{4}}-1}(1-m_{Na})-0.28\frac{V_{a}-V_{t}-40}{e^{\frac{V_{a}-V_{t}-40}{5}}-1}m_{Na}$$

$$\frac{\partial h_{Na}}{\partial t}=0,128e^{\frac{17+V_{t}-V_{a}}{18}}(1-h_{Na})-\frac{4h_{Na}}{1+e^{2(40+V_{s}-{Va}_{})}}$$

$$\frac{\partial m_{Kd}}{\partial t}=0.16\left(\frac{V_{t}-V_{a}+20}{e^{\frac{V_{t}-V_{a}+20}{5}}-1}\right)(1-m_{Kd})-0.25e^{\frac{20+V_{t}-V_{a}}{40}}m_{Nd}$$

$$\frac{\partial m_{KCa}}{\partial t}=\frac{3}{1+e^{\frac{0.08-[Ca]}{0.8}}}(1-m_{KCa})-20m_{KCa}$$ (3)

*V*_*t*_ is a threshold membrane potential (Table 1) and [Ca] is the calcium concentration. For all cells, intracellular Ca^2+^ dynamics was described by a simple first-order model (4).


$$\frac{\partial [Ca]}{\partial t}=0.001(-0.35I_{Ca}-MU[Ca]+0.04{MU}^{2})$$ (4)

*MU* is the calcium dynamic dissipation. See [43,98] for more detail and previous implementations of Eqs (3) and (4).

### GABA synaptic currents

In the presented model the only one connection considered between neurons were GABA dependent synaptic currents. We studied the action of both, GABA-A and GABA-B receptors dependent currents. Both are the result of LN activity but each one has its own dynamics. The synaptic current *I*_*syn*_ was calculated as the summation of GABA currents *I*_*GABA-A*_ and *I*_*GABA-B*_.


$$I_{syn}=I_{GABA‐A}+I_{GABA‐B}$$

The GABA-A dependent current for the neuron *i* (*I*_*GABA-A*_^*i*^) was given by the Eq (5)

$$I_{GABA-A}^{i}=\underset{k}{\sum }g_{GABA-A}^{ik}m_{GABA-A}^{k}(V_{s}^{i}-V_{GABA})$$ (5)

where *k* represents the LN that synapses with the *i* neuron and g_*GABA-A*_^*ik*^ is the strength of the connection as in the Fig 2A–2C (for example, 0 if the *i* neuron was not connected with the *k* neuron). The gating variable *m*_*GABA-A*_ was varied in time and depended on its own dynamic Eq (6) and *V*_*GABA*_ is the resting membrane potential for GABA (value in Table 1).


$$\frac{\partial m_{GABA-A}}{\partial t}=K_{fGABA-A}(1-m_{GABA-A})\frac{1}{1+e^{\frac{30-V_{a}}{2}}}K_{rGABA-A}m_{GABA-A}$$ (6)

The GABA-B current for the neuron *i* (*I*_*GABA-B*_^*i*^) was given by the equation

$$I_{GABA-B}^{i}=\underset{k}{\sum }g_{GABA-A}^{ik}\left(V_{s}^{i}-V_{GABA}\right)\frac{m_{GABA-B}^{k}}{m_{GABA-B}^{k}+K_{dGABA-B}}$$ (7)

where *k* indicates the LN neuron that synapses with the *i* neuron. g_GABA-B_^ik^ is the strength connection setting as in Fig 2A–2C. The gating variable *m*_*GABA-B*_ is a temporal variable with its own dynamic Eq (8) and K_dGABA-B_ is a constant.

$$\frac{\partial h_{GABA-B}}{\partial t}=K_{1GABA-B}(1-h_{GABA-B})\frac{1}{1+e^{\frac{-V_{a}}{2}}}-K_{2GABA-B}h_{GABA-B}$$

$$\frac{\partial m_{GABA-B}}{\partial t}=K_{3GABA-B}h_{GABA-B}-K_{4GABA-B}m_{GABA-B}$$ (8)

where *h*_*GABA-B*_ is an internal temporal variable.

### Similarity between imaging and model outputs

We defined a similarity index to quantify the extent to which the response pattern generated by the AL model replicates a standard response pattern measured from the real AL. The index was conceived to capture the complexity of the pattern in terms of the relative amplitude of the different glomeruli and their temporal profiles.

First we established an odor elicited activity pattern measured from the real AL that could be used as a standard pattern. We took all recordings from bees in which we had identified the same set of glomeruli and for each identified glomerulus we averaged across animals the response elicited upon 4 seconds stimulation with 2-octanone (dilution 1/100 in liquid phase). This average response pattern was taken as a standard calcium pattern elicited by the odor.

For the model output we took the intracellular calcium concentrations determined for each of the three PNs in one glomerulus and averaged them to obtain a single calcium signal that represented the glomerulus response as a whole. Next, each glomerular calcium trace from the model was binned to 8 frames per second to allow direct frame by frame comparison with the calcium imaging recordings. This procedure was made for every PN and glomerulus in the model, thus, at the end we obtained 20 calcium traces that together represented the spatio-temporal code elicited by the stimulation.

Afterwards, we calculated the Pearson´s correlation coefficient between each of the standard calcium traces and each of the 20 traces computed from the model. The highest correlation coefficient obtained was taken as the best match between a measured and a model calcium trace. This correlation value was saved and the respective pair of traces was removed to calculate the next best match between the remaining model and measured calcium traces. This procedure was repeated until having paired each of the recorded glomeruli to one of the model traces. Finally, all correlation values were averaged and this average was taken as a similarity index between the behavior of the actual AL and the model AL.

## Supporting information

S1 Fig Temporal detail of GABA-A and GABA-B receptors dependent inhibition in projection neurons of the honey bee antennal lobe (discriminated by glomeruli). Odor-elicited activity was measured in uniglomerular projection neurons (uPNs) by means of calcium imaging. Three groups of bees were measured first under physiological saline solution and then under perfusion with picrotoxin to block GABA-A receptors (brown traces; N(bees) = 8), or CGP54626 to block GABA-B receptors (blue traces; N(bees) = 9) or both blockers together (purple trace; N(bees) = 9). The activity measured under saline condition was subtracted from the activity measured under the indicated blocker. Before subtraction, the activity was normalized within each bee by setting to 1 the highest activity measured in each bee. The graphs show the mean and SEM of the subtraction across bees with the same treatment. The number on top of each graph indicates the glomerulus identified across all bees according to anatomical and functional honey bee AL atlas [66,48,99]. As observed, the temporal profile of the GABAergic inhibition that is evidenced after the three different treatments is very consistent across glomeruli. The black bar at the bottom of each graphs indicate the 4-seconds pulse of 2-octanone.
(PDF)

S2 Fig Principal component analysis to represent the spatio-temporal patterns of activity elicited by different concentrations of 2-octanone (control and GABA blocked). Gray to black traces correspond to the activity patterns measured under perfusion with physiological saline solution. Pink to purple traces correspond to the same animal and to activity patterns measured under perfusion with the cocktail PTX + CGP. The trajectories plotted in the principal component space correspond to activity measured from 250 ms before odor onset to 2500 ms after odor onset. Odor concentrations are indicated by the corresponding dilution of the odorant in mineral oil (V/V). The headspace of the solution was used as stimulus. Notice that activity patterns elicited in control (saline) conditions project towards the same direction of the PC space which is consistent with high correlation coefficients between activity patterns elicited by different concentrations (Fig 5). In contrast, blocking GABA receptors generate a much broader distribution of the trajectories. As it is shown, the different concentrations spread along two orthogonal components of the PCA space (97% of variance explained by the first two PCs). This broader distribution explains the lower correlation coefficients between the spatio-temporal patterns when GABA receptors were blocked (Fig 5).
(PDF)

S3 Fig Odorant dilution in liquid phase (2-octanone diluted in mineral oil) and its concentration in the headspace. One ml of the headspace contained in saturated 6ml vials were injected into gas chromatograph attached to a flame ionization detector to quantify odor concentration in the headspace. As observed the headspace provides a continuous graded concentration along the range used.
(PDF)
