# Supplementary material for: Gain modulation and odor concentration invariance in early olfactory networks
Source: PLoS Comput Biol. 2023 Jun 21;19(6):e1011176. doi: 10.1371/journal.pcbi.1011176 (PMC10317235; doi:10.1371/journal.pcbi.1011176)
Supplement: S1 Fig — Odor-elicited activity was measured in uniglomerular projection neurons (uPNs) by means of calcium imaging. Three groups of bees were measured first under physiological saline solution and then under perfusion with picrotoxin to block GABA-A receptors (brown traces; N(bees) = 8), or CGP54626 to block GABA-B receptors (blue traces; N(bees) = 9) or both blockers together (purple trace; N(bees) = 9). The activity measured under saline condition was subtracted from the activity measured under the indicated blocker. Before subtraction, the activity was normalized within each bee by setting to 1 the highest activity measured in each bee. The graphs show the mean and SEM of the subtraction across bees with the same treatment. The number on top of each graph indicates the glomerulus identified across all bees according to anatomical and functional honey bee AL atlas [66,48,99]. As observed, the temporal profile of the GABAergic inhibition that is evidenced after the three different treatments is very consistent across glomeruli. The black bar at the bottom of each graphs indicate the 4-seconds pulse of 2-octanone. (PDF) [file pcbi.1011176.s001.pdf]

S1 fig

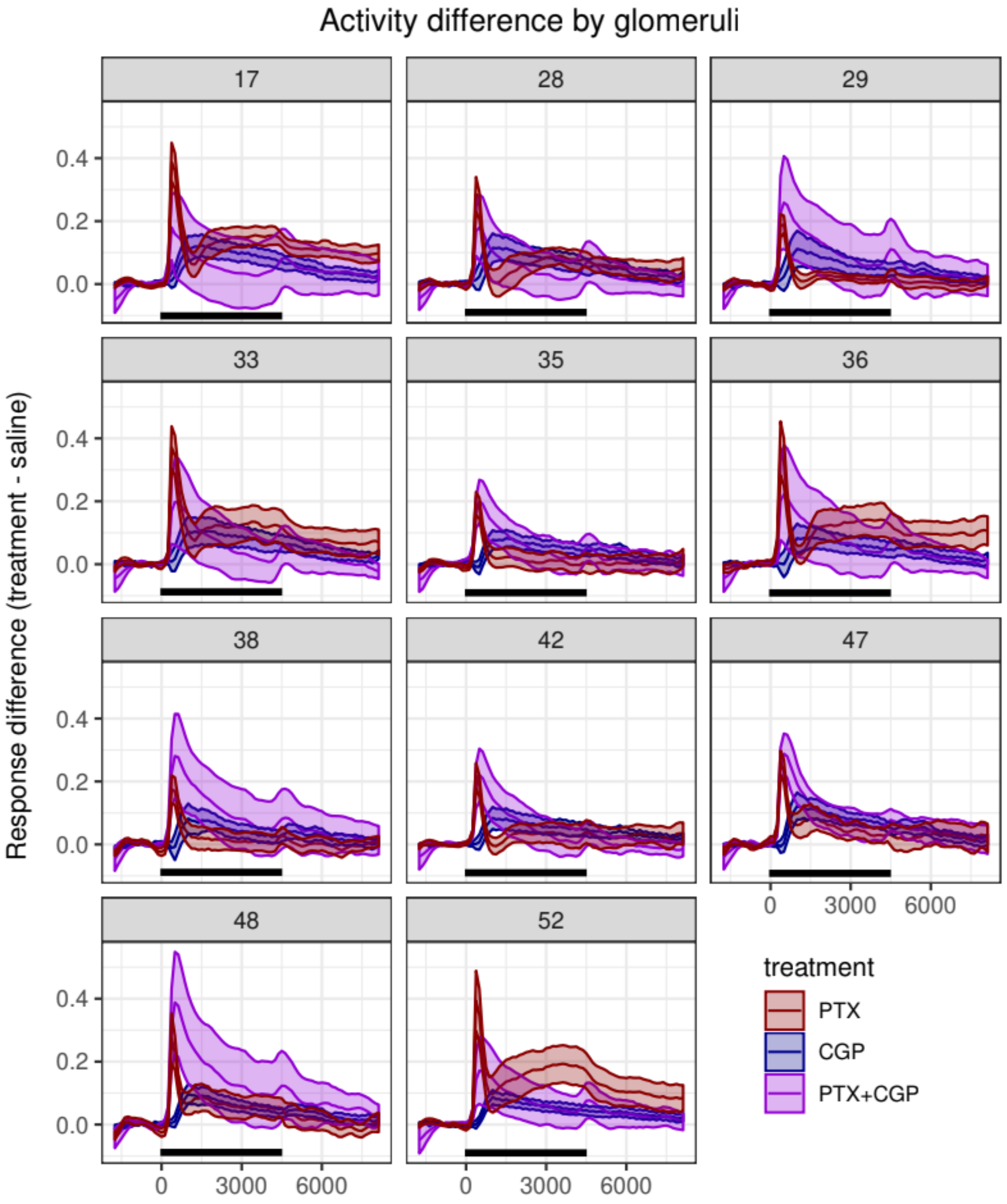

Temporal detail of GABA-A and GABA-B receptors dependent inhibition in projection neurons of the honey bee antennal lobe (discriminated by glomerulus)

Odor-elicited activity was measured in uniglomerular projection neurons (uPNs) by means of calcium imaging. Three groups of bees were measured first under physiological saline solution and then under perfusion with picrotoxin to block GABA-A receptors (red traces; N = 9), or CGP54626 to block GABA-B receptors (blue traces; N= 8) or both blockers together (purple trace; N = 7). The activity measured under saline condition was subtracted frame by frame from the activity measured under the indicated blocker. Before subtraction, the activity was normalized within each bee setting to 1 the highest activity measured in each bee. The graphs show the mean and SEM of the subtraction across bees with the same treatment. The number on top of each graph indicates the glomerulus identified across all bees according to anatomical and functional honey bee AL atlas (Flanagan & Mercer, 1989; Galizia, McIlwrath, et al., 1999; Galizia, Sachse, Rappert, & Menzel, 1999). As observed, the temporal profile of the GABAergic inhibition that is evidenced after the three different treatments is very consistent across glomeruli. The black bar at the bottom of each graphs indicates the 4-seconds pulse of 2-octanone.
