# Supplementary material for: Gain modulation and odor concentration invariance in early olfactory networks
Source: PLoS Comput Biol. 2023 Jun 21;19(6):e1011176. doi: 10.1371/journal.pcbi.1011176 (PMC10317235; doi:10.1371/journal.pcbi.1011176)
Supplement: S2 Fig — Gray to black traces correspond to the activity patterns measured under perfusion with physiological saline solution. Pink to purple traces correspond to the same animal and to activity patterns measured under perfusion with the cocktail PTX + CGP. The trajectories plotted in the principal component space correspond to activity measured from 250 ms before odor onset to 2500 ms after odor onset. Odor concentrations are indicated by the corresponding dilution of the odorant in mineral oil (V/V). The headspace of the solution was used as stimulus. Notice that activity patterns elicited in control (saline) conditions project towards the same direction of the PC space which is consistent with high correlation coefficients between activity patterns elicited by different concentrations (Fig 5). In contrast, blocking GABA receptors generate a much broader distribution of the trajectories. As it is shown, the different concentrations spread along two orthogonal components of the PCA space (97% of variance explained by the first two PCs). This broader distribution explains the lower correlation coefficients between the spatio-temporal patterns when GABA receptors were blocked (Fig 5). (PDF) [file pcbi.1011176.s002.pdf]

**S2 fig**

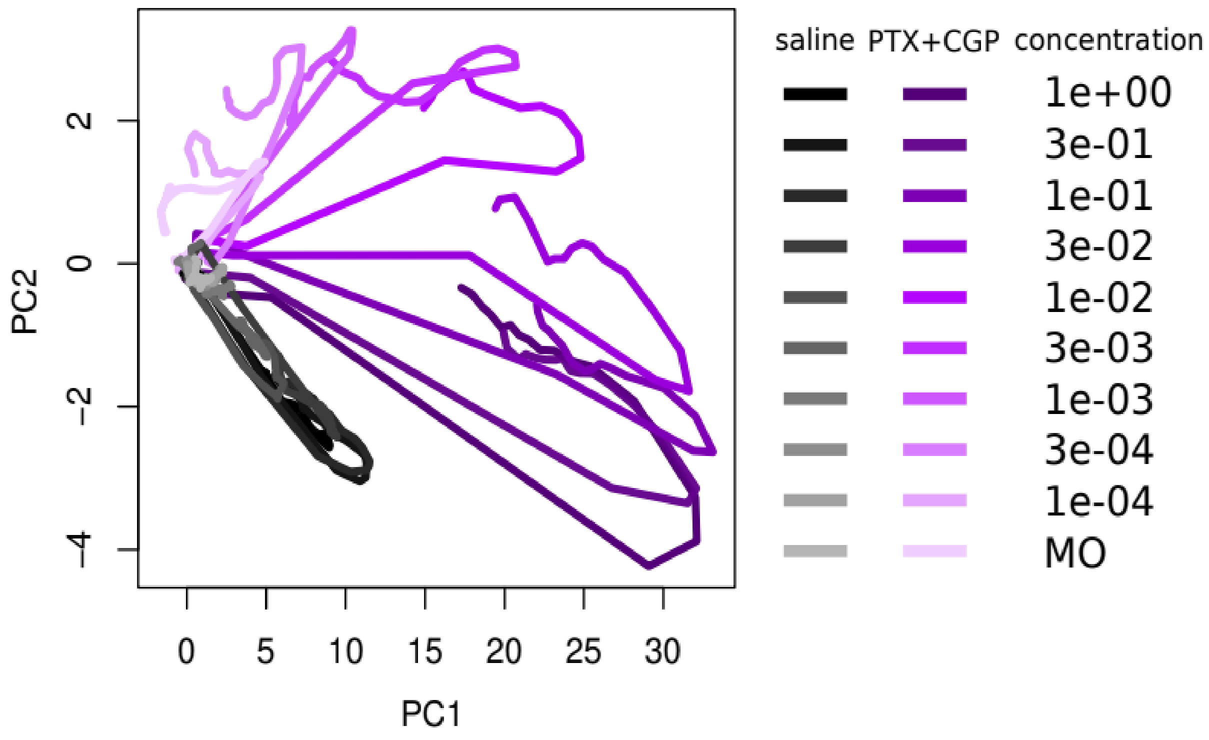


**Principal component analysis to represent the spatio-temporal patterns of activity elicited by different concentrations of 2-octanone (control and GABA blocked).**

Gray to black traces correspond to the activity patterns measured under perfusion with physiological saline solution. Pink to purple traces correspond to the same animal and to activity patterns measured under perfusion with the cocktail PTX + CGP. The trajectories plotted in the principal component space correspond to activity measured from 250 ms before odor onset to 2500 ms after odor onset. Odor concentrations are indicated by the corresponding dilution of the odorant in mineral oil. The headspace of the solution was used as stimulus.

Notice that activity patterns elicited in control (saline) conditions project towards the same direction of the PC space which is consistent with high correlation coefficients among pairs of measurements (figure 5). In contrast, blocking GABA receptors generates a much broader distribution of the trajectories. PCA: 94.3% and 3.6% of variance explained by PC1 and PC2 respectively. The broader distribution explains the lower correlation coefficients between the spatio-temporal patterns when GABA receptors were blocked (figure 5).
