# Supplementary material for: Gain modulation and odor concentration invariance in early olfactory networks
Source: PLoS Comput Biol. 2023 Jun 21;19(6):e1011176. doi: 10.1371/journal.pcbi.1011176 (PMC10317235; doi:10.1371/journal.pcbi.1011176)
Supplement: S3 Fig — One ml of the headspace contained in saturated 6ml vials were injected into gas chromatograph attached to a flame ionization detector to quantify odor concentration in the headspace. As observed the headspace provides a continuous graded concentration along the range used. (PDF) [file pcbi.1011176.s003.pdf]

S3 fig

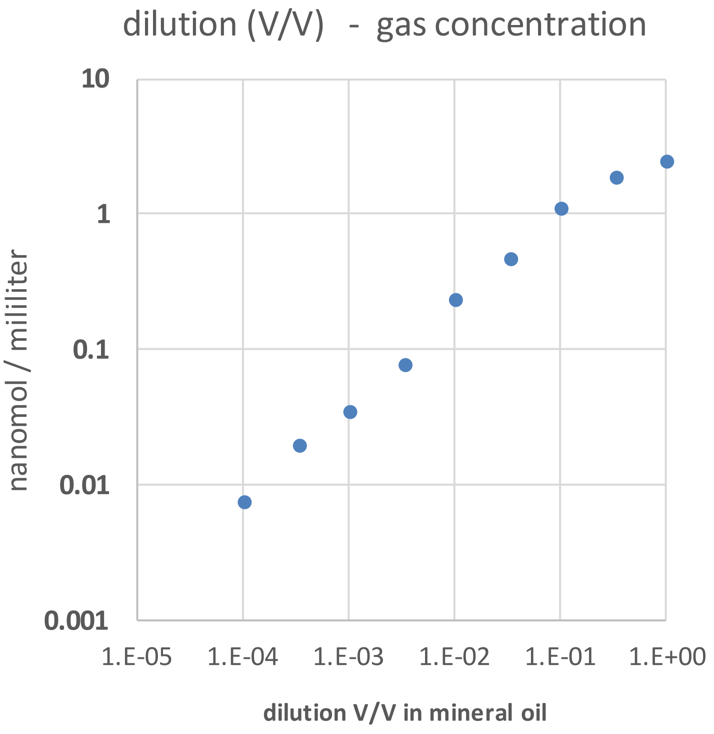

Odorant dilution in liquid phase (2-octanone diluted in mineral oil) and its concentration in the headspace. One ml of the headspace contained in saturated 6ml vials were injected into gas chromatograph attached to a flame ionization detector to quantify odor concentration in the headspace. As observed the headspace provides a continuous graded concentration along the range used.
